# Process optimization and kinetic study of biodegradation of dimethyl phthalate by KS2, a novel strain of *Micrococcus* sp.

**DOI:** 10.1038/s41598-023-29256-x

**Published:** 2023-03-08

**Authors:** Sangram Shamrao Patil, Hara Mohan Jena

**Affiliations:** Department of Chemical Engineering, National Institute of Technology, Rourkela, Odisha 769008 India

**Keywords:** Applied microbiology, Bacteriology, Environmental microbiology, Industrial microbiology, Chemical engineering

## Abstract

The present study elucidates identification and characterization of dimethyl phthalate (DMP) degrading novel bacterial strain, *Micrococcus* sp. KS2, isolated from soil contaminated with municipal wastewater. Statistical designs were exercised to achieve optimum values of process parameters for DMP degradation by *Micrococcus* sp. KS2. The screening of the ten important parameters was performed by applying Plackett–Burman design, and it delivered three significant factors (pH, temperature, and DMP concentration). Further, response surface methodology involving central composite design (CCD) was implemented to examine mutual interactions among variables and achieve their optimal response. The predicted response indicated that maximum DMP degradation (99.67%) could be attained at pH 7.05, temperature 31.5 °C and DMP 289.19 mg/l. The strain KS2 was capable of degrading up to 1250 mg/l of DMP in batch mode and it was observed that oxygen was limiting factor in the DMP degradation. Kinetic modeling of DMP biodegradation indicated that Haldane model fitted well with the experimental data. During DMP degradation, monomethyl phthalate (MMP) and phthalic acid (PA) were identified as degradation metabolites. This study provides insight into DMP biodegradation process and proposes that *Micrococcus* sp. KS2 is a potential bacterial candidate to treat effluent containing DMP.

## Introduction

Phthalate esters (PEs) are considered one of the most common organic pollutants due to their extensive occurrence in soils, sediments, natural water resources, plant tissue, landfill leachates, and aquatic biota^1,2^. Dimethyl phthalate (DMP) is the low molecular weight phthalate ester used in plastic products to increase their flexibility, pellucidity, resilience, and longevity^3,4^. They are also used in cosmetics, toys, adhesives, fiberglass, surface coatings manufacturing, paints, inks, and in insect repellents^5^. Phthalates persist in the environment with a half-life of ≈ 20 years and especially, aqueous hydrolysis half-lives for DMP are 3.2 years^3,6^. DMP is known to be a subchronic toxicant and an endocrine-disrupting chemical (EDC) which affects both male and female reproductive systems. Also, it is a teratogen, hampering the development of the fetus^7–9^. Hence, wastewater-containing DMP needs to be treated before being discharged into the environment.

From the past years, various researchers reported the application of indigenous microorganisms for biological treatment of wastewater in an industrial viewpoint. This encourages isolating, identifying and characterizing indigenous microorganisms for biological treatment of wastewaters. Large numbers of reports are available on the biodegradation of phthalates by bacteria and their characterization^10–14^. Reported biodegradation studies of DMP is summarized in Table 1. In the previous studies, *Micrococcus* sp. is reported as one of the most promising bacteria for PEs biodegradation and they are mainly focused on the identification and characterization of microbes^15,16^. However, until now no attempt was made to optimize DMP biodegradation by *Micrococcus* sp. using statistical design and the present work is first to execute the same. Besides, there is no report available on the kinetic study using various models, metabolite identification and estimation of dissolved oxygen profile of DMP biodegradation by *Micrococcus* sp.

**Table 1:** Biodegradation studies dimethyl phthalate (DMP) reported in literature.

Microorganism	Isolated from	Phthalate ester	Initial concentration	Performance (% degradation and treatment time)	References
*Sphingomonas paucimobilis, Xanthomonas maltophilia*	Sewage sludge	DMP	400 mg/l	100% in 96 h	Wang et al.^17^
*Pseudomonas fluorescens* FS1	Activated sludge obtained from a petrochemical factory	DMP	400 mg/l	> 99% in 96 h	Zeng et al.^18^
*Burkholderia cepacia* DA2	Deep-ocean sediment	DMP	400 mg/l	100% in 216 h	Wang et al.^19^
*Rhodococcus ruber*	Activated sludge collected from a dyeing plant	DMP	200 mg/l	75% in 144 h	Lu et al.^4^
*Bacillus* *subtilis*	Soil	DEP	50 mg/l	> 95% in 24 h	Navacharoen and Vangnai^20^
*Variovorax* sp.	Electronic and plastic wastes contaminated soil	DMP	300 mg/l	> 99% in 30 h	Prasad and Suresh^21^
*Arthrobacter* sp. ZH2	Mangrove sediment	DMP	200 mg/l	100% in 40 h	Wang et al.^22^
*Xenorhabdus indica*	*Entomopathogenic nematode*	DMP	400 mg/l	98.75% in 12 days	Pranaw et al.^23^
*Bacillus* *thuringiensis*	Cotton field soil	DMP	400 mg/l	100% in 72 h	Surhio et al.^6^
*Bacillus* *subtilis*	Soil	DEP	400 mg/l	97% in 48 h	Sompornpailin et al.^24^
*Bacillus* sp. QD14	Soil sample collected from the river	DMP	100 mg/l	100% in 64 h	Mo et al.^25^

The objective of the present research was to isolate and identify a phthalate-degrading bacterium from contaminated soil at municipal wastewater discharge site. It was also projected to investigate the individual and the interactive effects of process parameters for DMP degradation and obtain optimum values of the significant factors. The investigation was also intended to study the growth kinetics, dissolved oxygen profile and identify degradation intermediate.

## Materials and methods

### Materials

Dimethyl phthalate (DMP) with 99% purity was procured from Sigma-Aldrich Chemicals and used as a source of carbon throughout the present work. All other chemicals of analytical reagent grade were obtained from Merck®, India and HIMEDIA®, India.

### Isolation and identification of DMP degrading bacteria

DMP degrading efficient bacterial strain was isolated from soil samples collected from municipal wastewater discharge site at Koel River, Rourkela (Odisha, India). The acclimatized strains were individually screened at higher concentrations of DMP to get efficient DMP degrading bacterium. Among them, the strain (KS2) exhibited tolerance at high concentrations of DMP, was selected as a subject of the present study. Strain (KS2) was inspected for colony morphology: size, shape, color, margin, opacity, elevation, and texture. Gram staining, Motility test, and biochemical tests were performed for morphological and biochemical characterization. For examining cell shape, Scanning Electron Microscopy (SEM) was executed using scanning electron microscope (JEOL, Japan). For 16S rRNA sequencing, the strain was cultured in Luria–Bertani (LB) medium for 30 h, and its genomic DNA was extracted and further processed as per standard procedure^26^.

### Medium conditions

The isolate was preserved on nutrient agar slants and subcultured every two weeks. A fresh bacterium culture developed on nutrient agar was incubated in nutrient broth at 120 rpm and 30 °C for 30 h^27^. Then, this culture was used as inoculum throughout the study. Mineral salt media (MSM) used for in this study has a composition as follows (g/l): 4 (NH_4_)_2_SO_4_, 4 KH_2_PO_4_, 6 Na_2_HPO_4_, 0.2 MgSO_4_.7H_2_O, 0.1 yeast extract, 0.01 CaCl_2_.H_2_O, 0.01 FeSO_4_.7H_2_O and pH maintained at 7^28^. DMP was used an only carbon source throughout the study.

### Design of experiments and statistical analysis

#### Identification of significant variables by Plackett–Burman design

Screening of the ten important parameters [pH, temperature, DMP concentration, inoculum size, (NH_4_)_2_SO_4_, KH_2_PO_4_, Na_2_HPO_4_, MgSO_4_, Yeast extract, agitation speed] for DMP degradation was performed using Plackett–Burman design (PBD). The variables were considered at face-centered positions (− 1, 0, + 1). The first order polynomial model for PBD analysis was:

1 $$\user2{Y } = {\varvec{R}}_{0} + \user2{ }\sum {\varvec{R}}_{{\user2{i }}} {\varvec{X}}_{{\varvec{i}}}$$

The set of 16 runs was designed, and the response was determined for each run independently. The regression analyzed factors with confidence level above 95% (*p* values < 0.05) were suggested to have the substantial effect on degradation of DMP and further proposed for optimization by central composite design.

#### RSM based full factorial central composite design (CCD)

The full factorial CCD was performed to examine the influence of three significant parameters and to obtain interactions among them. The variables were considered at three different levels (− 1, 0, + 1) independently and the insignificant factors were set at their center level throughout the present investigation. The quadratic equation was applied to study the experimental data is as follows:

2 $${\varvec{Y}} = \user2{ R}_{0} \user2{ } + \user2{ }\mathop \sum \limits_{{{\varvec{i}} = 1}}^{{\user2{ n}}} {\varvec{R}}_{{\varvec{i}}} {\varvec{X}}_{{\varvec{i}}} + \user2{ }\mathop \sum \limits_{{{\varvec{i}} = 1}}^{{\varvec{n}}} {\varvec{R}}_{{{\varvec{ii}}}} {\varvec{X}}_{{\varvec{i}}}^{{2\user2{ }}} \user2{ } + \mathop \sum \limits_{{{\varvec{i}} = 1}}^{{\varvec{n}}} \mathop \sum \limits_{{{\varvec{j}} = 1}}^{{\varvec{n}}} {\varvec{R}}_{{{\varvec{ij}}}} {\varvec{X}}_{{\varvec{i}}} {\varvec{X}}_{{\varvec{j}}}$$

#### Statistical analysis and experimentation

The developed model was statistically assessed using analysis of variance (ANOVA). It entitles the correlation between the response and significant variables, and it comprises F value, *p* value (probability), and the coefficient of determination (R^2^) which defines the fit of the regression model. The three-dimensional surface plots of model predicted DMP degradation (%) were developed to estimate the effect of variables on the response. Design-Expert—10.0.0, (Stat-Ease Inc., Minneapolis, USA) (Trial version) was used to design and analysis of experimentations. The optimization runs were executed in mineral salt media for 48 h in triplicates. The culture samples were retrieved at 6 h intervals and analyzed for residual DMP concentration.

### DMP biodegradation and identification of intermediates

Batch biodegradation experiments were performed at optimized conditions by varying DMP concentration from 50 to 1250 mg/l. Erlenmeyer flasks (250 ml) containing 100 ml of MSM (pH 7.05) were inoculated with overnight grown culture and incubated at 31.5 °C and 120 rpm in an orbital shaker. Batch experiments were exercised in triplicate for each DMP concentration. The 2 ml of samples were removed initially at an interval of 6 h and then 12 h interval until complete degradation occurred and were analyzed for cell growth and residual DMP concentration.

For analysis of intermediates produced during the degradation process, supernatant from the samples was extracted with n-Hexane and subsequently evaporated to dryness at 30 °C. The dried samples were mixed with methanol and filtered through a syringe filter (pore size 0.22 µm). These samples (1 ml) were subjected to Electrospray ionization-Mass spectrometry (ESI–MS, Perkin Elmer, USA). The ESI–MS spectra were recorded in the positive electron ionization (EI) mode; the drying gas temperature was 300 °C with capillary exit voltage of 100 V. The mass range was 120–400 mu and pulse counting was 100 µs with coarse resolution 10.9.

### Analytical methods

The culture samples were collected at 6 h interval and centrifuged at 10,000 rpm for 10 min at 30 °C. For cell growth determination, the pellet was transferred in distilled water and analyzed against a reference (distilled water) at 600 nm using a UV–visible spectrophotometer (Jasco V-530, Japan). The supernatant was extracted using n-hexane and further evaporated to dryness at 30 °C. The dried samples were mixed with methanol and then filtered through a syringe filter (pore size 0.22 µm). For the analysis of metabolites produced during the DMP degradation process, the aliquots were examined by gas chromatography-mass spectrometry (GC–MS) (Agilent Technologies, USA). The following GC–MS conditions were exercised: a DB-5 column (30 m × 0.25 mm × 0.25 m) with helium as carrier gas at a flow rate of 1.0 mL min^−1^ was used. Inlet temperature was set at 300 °C in split mode with a split ratio of 20:1. The GC oven temperature program was employed as follows: 50 °C hold for 1 min, raised at 10 °C min^−1^ to 180 °C and hold for 1 min, then at 20 °C min^−1^ to 280 °C and hold for 7 min (Liu et al., 2015). Mass spectra were attained in the electron ionization (EI) mode with a mass range of 50–500 amu. MS temperature was set at 230 °C (source) and 150 °C (Quad). The peaks of PEs and its degradation metabolites were obtained by analyzing the scan with the standard mass spectra peaks accessible through the library of the National Institute of Standards and Technology (NIST) (Agilent Technologies, USA).

### Study the dissolved oxygen (DO) profile in the course of DMP biodegradation

Providing sufficient oxygen to aerobic bacterial cultures is important factor for the efficient biodegradation. Aeration in shake flasks is attained by gas liquid contact owing to shaking the vessels in reciprocating or rotary shaking machines^29^. In the present study, dissolved oxygen content was evaluated for DMP degradation by *Micrococcus* sp. KS2. It was measured in wide mouthed bottles for degradation of 50–1250 mg/l of DMP using MSM as a growth media independently. Bottles were kept on magnetic stirrer at 1000 rpm with immersed Dissolved Oxygen (DO) meter. Readings were taken at 0 h, during lag phase and at complete degradation of substrate.

### Cell growth kinetics

Growth kinetics of strain KS2 was performed by fitting the experimental data with Monod and Haldane model over the concentration range (0–1250 mg/l). Specific growth rate (μ) of the isolate at each DMP concentration was estimated using the Eq. (3):

3 $$\user2{\mu } = \user2{ }\frac{{{\mathbf{ln}}{\varvec{x}} - {\mathbf{ln}}{\varvec{x}}_{{\mathbf{0}}} }}{{\varvec{t}}}\user2{ }$$

In perspective of studying the non-inhibitory behaviour of DMP at low concentrations, Monod model was used in the present study. The Monod model equation is as follows:

4 $$\user2{ \mu } = \frac{{{\varvec{\mu}}_{{{\mathbf{max}}}} {\varvec{S}}}}{{{\varvec{Ks}} + {\varvec{S}}}}$$

DMP acts as inhibitory compound at high concentrations. Among several inhibitory models reported for growth kinetics of microbes, the Haldane model is mathematically simple and one of the prominent models to explore the substrate inhibition effect^30^. Hence, Haldane model was used to study of the growth kinetics of KS2 strain. The Haldane model equation is as follows:

5 $$\user2{ \mu } = \frac{{{\varvec{\mu}}_{{{\mathbf{max}}}} {\varvec{S}}}}{{{\varvec{Ks}} + {\varvec{S}} + \frac{{{\varvec{S}}^{2} }}{{{\varvec{K}}_{{\varvec{i}}} }}}}$$

The biokinetic parameters µ_max_, K_i_, and K_s_ were evaluated by fitting experimental growth data to kinetic models. Mathematical simulation software, MATLAB (V 8.5.1) was used to interpret the model using the non-linear regression method.

## Results and discussion

### Isolation and characterization of DMP degrading bacteria

**Figure 1:**
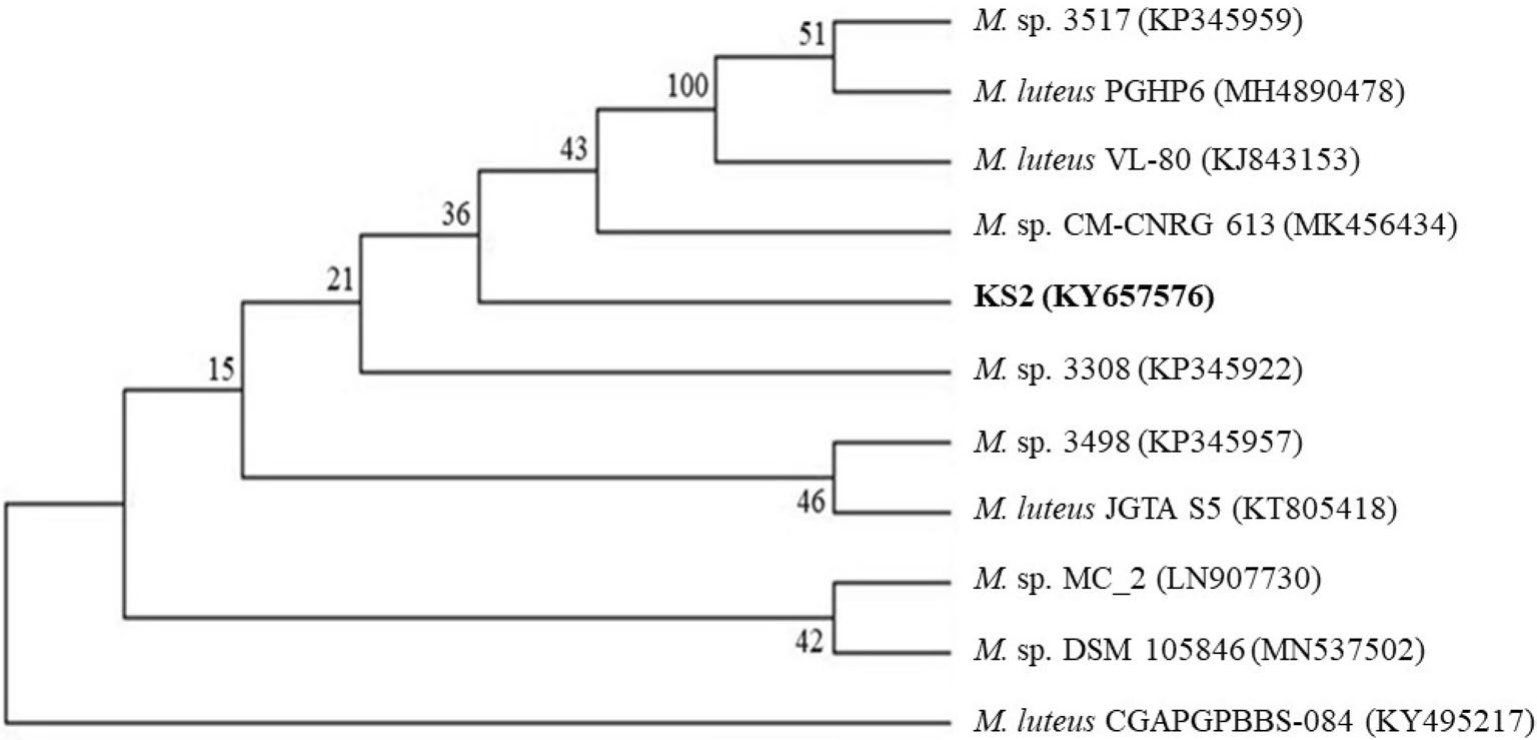
Phylogenetic tree obtained for strain KS2.

After the enrichment process, the bacterial strain (Strain KS2) tolerating high Dimethyl phthalate (DMP) concentration was isolated. Morphological characteristics of strain KS2 were observed by spread plate technique. The colony morphology of the isolate was circular, entire margin, and grey on nutrient agar plates. The morphological examination under the SEM showed strain was coccus in shape with a size of 1–1.5 µm. The isolate was identified as a Gram-positive, non-motile and aerobic. It showed positive catalase, oxidase, indole, urease, fructose fermentation, citrate, methyl red and Vogues-Proskauer tests. It exhibited negative results for nitrate reduction, glucose and lactose fermentation, gelatin liquefaction and starch hydrolysis tests. For molecular characterization, 16S rRNA sequence was determined, and BLAST was performed with GenBank database to compare with the available sequences. The phylogenetic tree indicated that strain KS2 belongs to the *Micrococcus* genus (Fig. 1). The obtained 16S rRNA sequence was registered and made accessible through GenBank (KY657576) of Nucleotide database of NCBI.

### Plackett–Burman design analysis

Using Plackett–Burman design (PBD), the effect of ten important variables on DMP degradation was studied. The following factors such as pH, temperature and DMP concentration were observed significant at the confidence level 95% and considered for the next step of optimization. Temperature and DMP concentration showed a positive effect while pH showed a negative effect on DMP degradation. The following model equation was obtained for DMP degradation (Y):

6 $$\begin{gathered} Y = 38.56{-}10.04X_{1} + 5.15X_{2} + 4.18X_{3} {-}0.17X_{4} + 1.63X_{5} \hfill \\ \quad \quad + 0.0258X_{6} {-}0.5492X_{7} {-}0.6958X_{8} + 0.0991X_{9} + 0.7958X_{10} \hfill \\ \end{gathered}$$

The R^2^ (Correlation coefficient) value of 0.98, specified that up to 98% variability in DMP degradation can be considered. The Predicted R^2^ (0.90) and Adjusted R^2^ (0.95) found to be reasonably close with each other confirming the model validity.

### Response surface methodology

A response surface design is suitable to implement if the optimal region for the process was recognized. Central composite design estimated interactions between pH, temperature, and DMP concentration obtained after screened using PBD. Regression analysis and Analysis of Variance (ANOVA) were defined the significance of model equation. The model terms with *p* value was less than 0.05 were considered as significant. The high F-value for the model (31.54) and nonsignificant F-value for lack of fit (2.38) confirmed the good fit of model. The predicted R^2^ (0.88) and adjusted R^2^ (0.93) were found to be in reasonable agreement. A coefficient of variation was found 6.87%, and it pointed towards the reliability of executed experimentations. The regression analysis of the DMP degradation (%, experimental) developed the second order polynomial equation that predicts the degradation process within the scope of present conditions and describes its biodegradation efficiency. It is represented as follows:

7 $$\begin{gathered} Y = 95.15 + 4.33X_{1} + 4.08X_{2} + 4.72X_{3} {-}4.80X_{1} X_{2} + 7.52X_{1} X_{3} \hfill \\ \quad \quad + 7.77X_{2} X_{3} {-}19.70X_{1}^{2} {-}5.37X_{2}^{2} {-}6.90X_{3}^{2} \hfill \\ \end{gathered}$$

The R^2^ (Correlation coefficient) value for regression model was 0.97; suggesting the quadratic model best fitted experimental results. The developed model was acceptable since regression equation accurately followed the experimental results.

**Figure 2:**
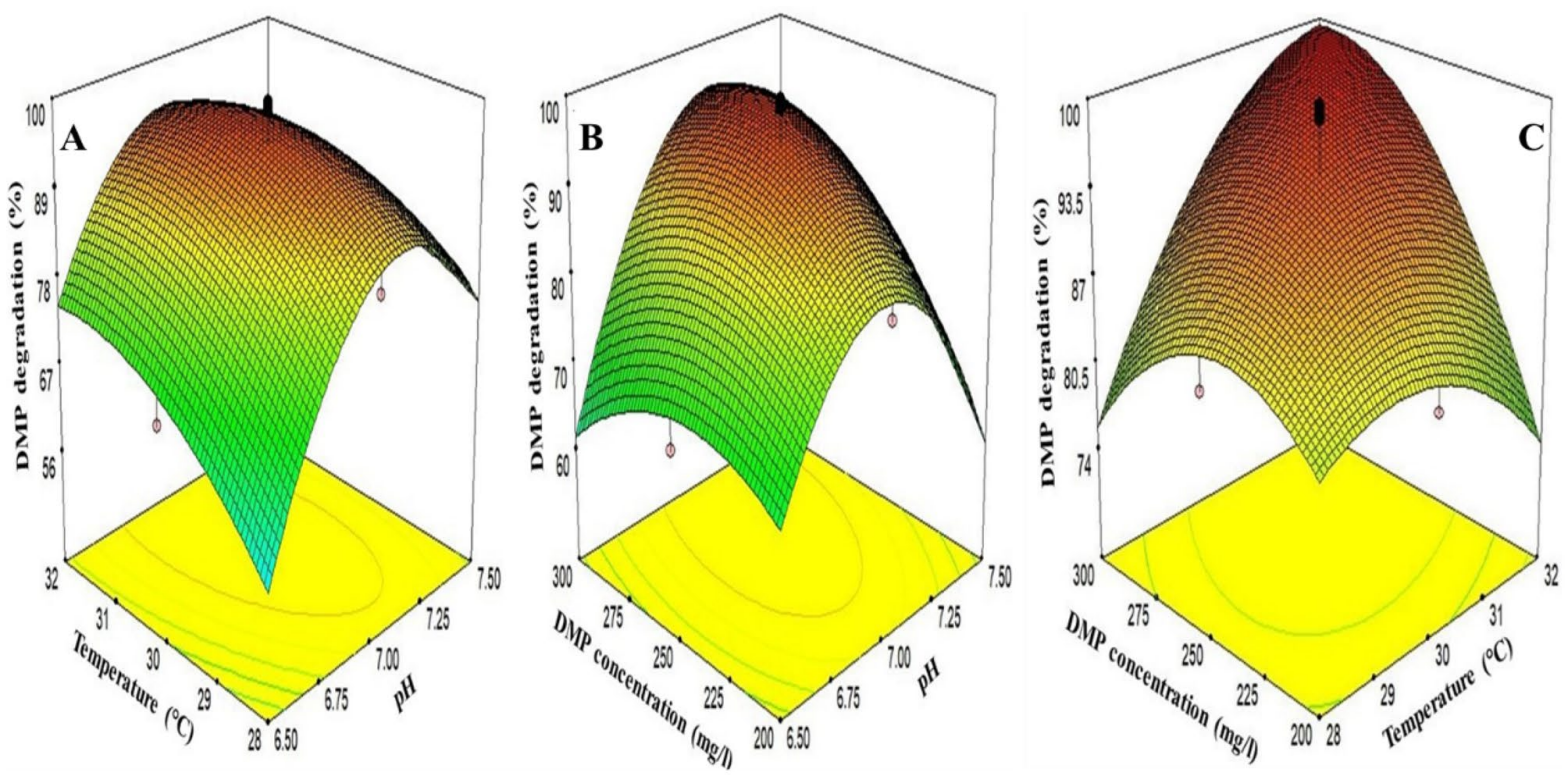
Three-dimensional response surface showing interactions between (**A**) temperature and pH (**B**) DMP concentration and pH (**C**) DMP concentration and temperature on DMP degradation by *Micrococcus* sp. KS2 while DMP concentration at Zero level.

Three-dimensional surface graphs were plotted to represent the behavior of the DMP biodegradation within the experimental design (Fig. 2). These plots define the effect of two variables on the response (Y), keeping others at their zero level. Figure 2A demonstrates the interactive influence of pH (X_1_) and temperature (X_2_) with respect to DMP degradation at 250 mg/l of DMP concentration (X_3_). DMP degradation (Y) showed increment as pH and temperature increased up to an optimum value and thereafter it declined with further increase in pH and temperature. This behaviour may be attributed to concurrent variation in pH and temperature affects the microbial enzymatic reactions and hence, collapses cellular functions^31,32^. Mo et al.^25^ also reported the similar type of behavior by *Bacillus* sp. towards DMP degradation as pH and temperature varied. The respective two-dimensional contour plot exhibited an elongated pattern, suggesting that the significance of pH and temperature interaction with regard to response.

The interactive plot of pH (X_1_) and DMP concentration (X_3_) and the response (Y) with temperature (X_2_) set at its center level is presented in Fig. 2B. The pattern of graph, and probability value proposed the significant interaction between pH and DMP concentration. Pradeep et al.^33^ have identified the similar combined effect of pH and substrate [di(2-ethylhexyl) phthalate (DEHP)] concentration on degradation by *Achromobacter denitrificans* strain SP1. Effect of temperature (X_2_) and DMP concentration (X_3_) on the response (Y), with pH (X_1_), at zero level is shown in Fig. 2C. The Y showed increment as the concentration of DMP was varied from 200 to 289.19 mg/l and then swiftly reduced as DMP concentration was increased. The quick decrease in response as a DMP concentration was increased might be due to its substrate inhibition effect^34^. The increment of tepmerature from 28 to 31.5 °C, upsurged the response (Y) and further Y declined as the temperature was increased. This demonstrated the significant effect of mutual interaction between temperature and DMP.

### Experimental validation of developed model

The developed statistical model was validated by performing degradation experiments at pH 7.05, temperature 31.5 °C and DMP 289.19 mg/l. Under the optimized conditions, the predicted DMP degradation was 99.67%, and one obtained from the experiments was 99.62%. The reasonably proximity between the experimental and model predicted results confirmed the validity of the model.

### Batch biodegradation of DMP

**Figure 3:**
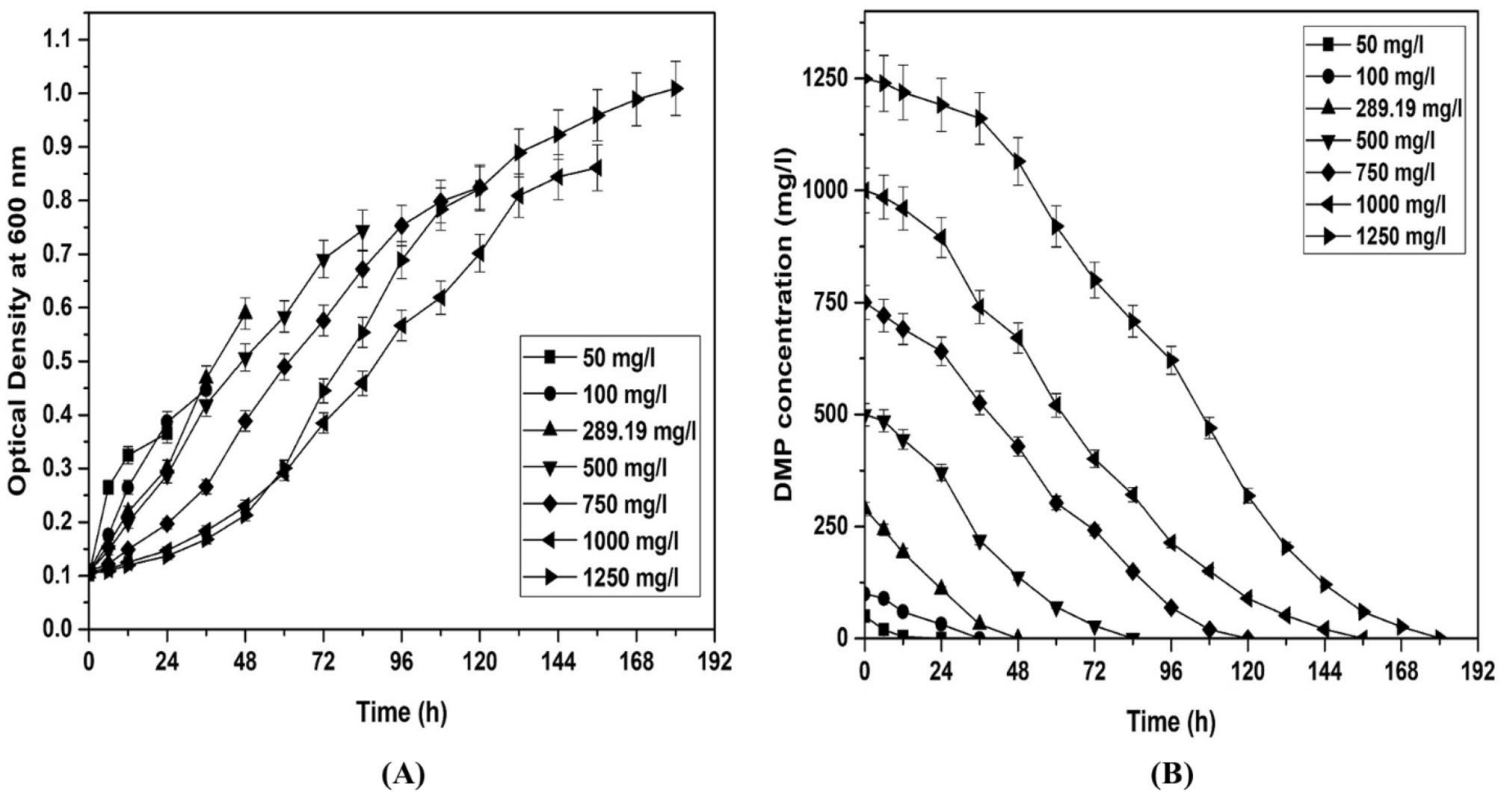
Cell growth (**A**) and DMP degradation behavior (**B**) of *Micrococcus* sp. KS2 at various DMP concentrations.

DMP degradation by *Micrococcus* sp. KS2 was studied at 50 to 1250 mg/l of DMP concentrations. Figure 3A represents growth profile for *Micrococcus* sp. KS2. No lag phase was observed up to optimized DMP concentration of 289.19 mg/l. The cell growth rate was found to be less at a low concentration up to 100 mg/l due to starvation. However, at higher DMP concentrations, a lag phase was observed, and the duration of lag phase was increased with the subsequent increase in DMP concentration. The occurrence of lag phase might be due to the toxicity of DMP. Similarly, Wang et al.^35^ reported biodegradation of DMP by *Burkholderia cepacia* DA2 and observed an increase in duration of lag phase with the subsequent increase in toxic effect of high DMP concentrations. Kaur et al.^36^ studied biodegradation of DMP (500 mg/l) and observed complete degradation in 72 h. Figure 3B represents DMP degradation by *Micrococcus* sp. KS2 at various initial DMP concentrations. It exhibited complete degradation of 50, 100, 289.19, 500, 750, 1000 and 1250 mg/l of DMP within 24, 36, 48, 84, 120, 156 and 180 h respectively. The degradation rate was higher at optimized DMP concentration (289.19 mg/l). As DMP concentration was increased beyond optimum value, the degradation rate was decreased as it acts as an inhibitory at high concentrations ^14^. In comparison with the DMP degradation rate reported for various bacterial strains, *Sphingomonas paucimobilis*, *Arthrobacter* sp., *Xenorhabdus indica*, *Acinetobacter* sp.^17,22,23,37^, *Micrococcus* sp. KS2 displayed competent degradation performance. Kaur et al.^36^ also studied biodegradation of DMP (500 mg/l) by *Bacillus marisflavi* RR014 and observed complete degradation in 72 h. However, they have not mentioned tolerance capacity of microbes. To the best of our knowledge, no study of DMP degradation by *Micrococcus* sp. under an aerobic condition at such high concentrations was reported. Thus, *Micrococcus* sp. KS2 presents high DEP degradation efficiency with a better growth rate.

### Analysis of degradation intermediates

**Figure 4:**
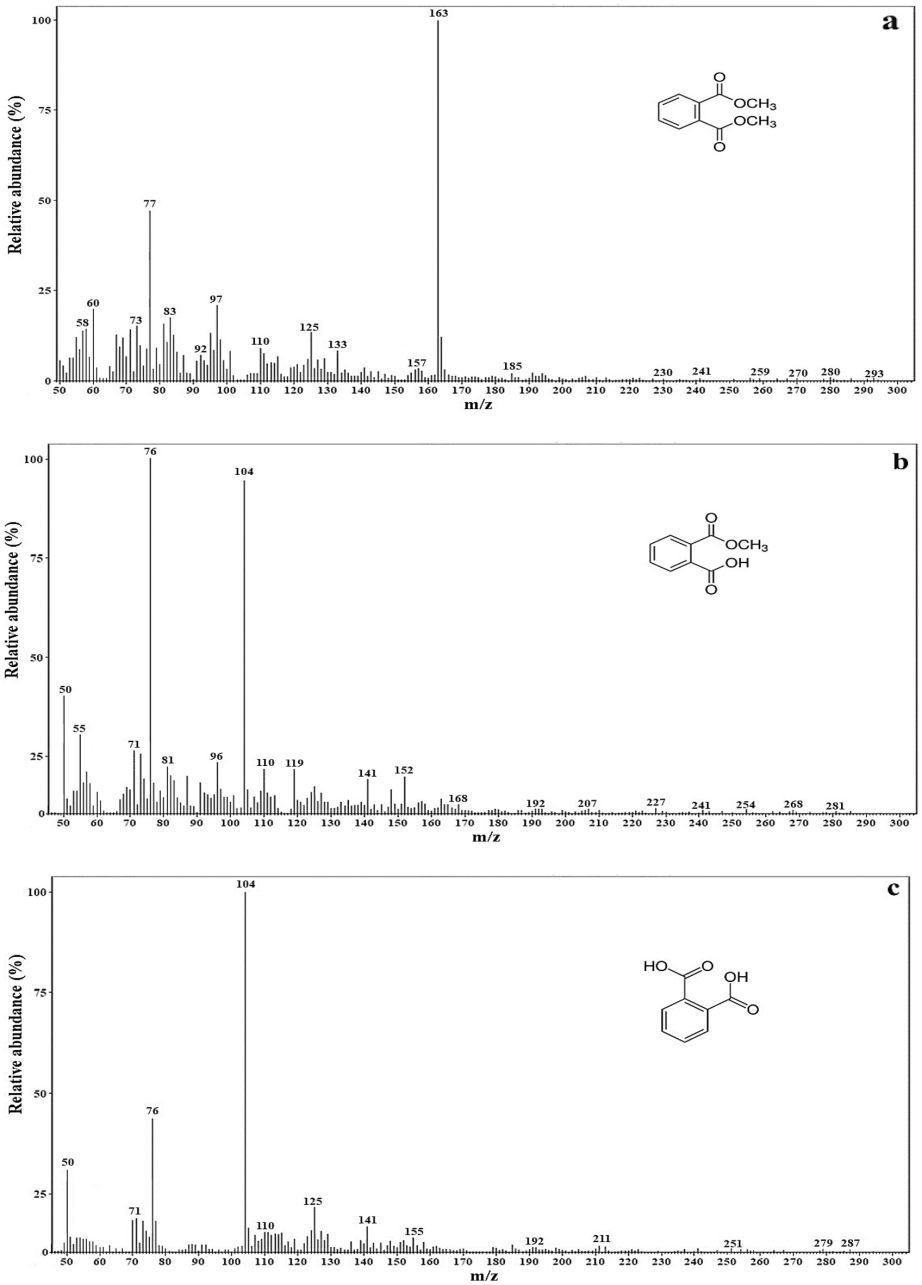
GC–MS fragmentation pattern representing the detection of DMP and its degradation metabolites for *Micrococcus* sp. KS2 [a DB-5 column (30 m × 0.25 mm × 0.25 m) with helium as carrier gas at a flow rate of 1.0 mL min^−1^; Inlet temperature 300 °C; split ratio of 20:1].

The intermediates formed during DMP degradation were determined based on the samples analyzed using GC–MS. Figure 4 shows the GC–MS fragmentation pattern for DMP degradation samples for *Micrococcus* sp. KS2. Table 2 represents the characterization of DMP degradation intermediates. After scanning the spectra with the standard peaks in the library, the characteristic ions of DMP, MMP for PA were observed. MMP and PA did not detect in an uninoculated DMP containing sterile medium. This indicates that isolate follow the de-esterification of DMP to MMP and then further hydrolysis to phthalic acid. Previously, Gu et al.^38^ studied biodegradation of DMP by *Sphingomonas yanoikuyae* DOS01 and reported that the biochemical pathway of DMP degradation involves the desertification of DMP to intermediate MMP, and then hydrolysis to PA before the further cleavage of an aromatic ring. Wang et al.^19^ studied biodegradation of DMP by *Burkholderia cepacia* DA2 and observed MMP and PA as metabolites of DMP biodegradation using HPLC analysis. In addition to these reports, the previously described biodegradation study of DMP also observed the hydrolysis of DMP to MMP and further conversion to PA^6,23,36^. Thus, the present study results confirm the occurrences of MMP and PA as DMP degradation metabolites. PA might be further converted to benzoic acid, which subsequently transformed to protocatechuate and eventually to CO_2_ and H_2_O through TCA (tricarboxylic acid) cycle^32^.

**Table 2:** Characterization of DMP degradation intermediates [DB-5 column (30 m × 0.25 mm × 0.25 m) with helium as carrier gas at a flow rate of 1.0 mL min^−1^; Inlet temperature 300 °C; split ratio of 20:1].

Strain	Compound	Chemical formula	Retention time (min)	Concentration (mg/l)	Major peaks (m/z)
*Micrococcus* sp. KS2	DMP	C_10_H_10_O_4_	8.03	92	163, 77, 97, 60
	MMP	C_9_H_8_O_4_	6.82	64	76, 104, 50, 55
	PA	C_8_H_6_O_4_	6.35	23	104, 76, 50, 125

### Study the dissolved oxygen variation during DMP degradation

**Figure 5:**
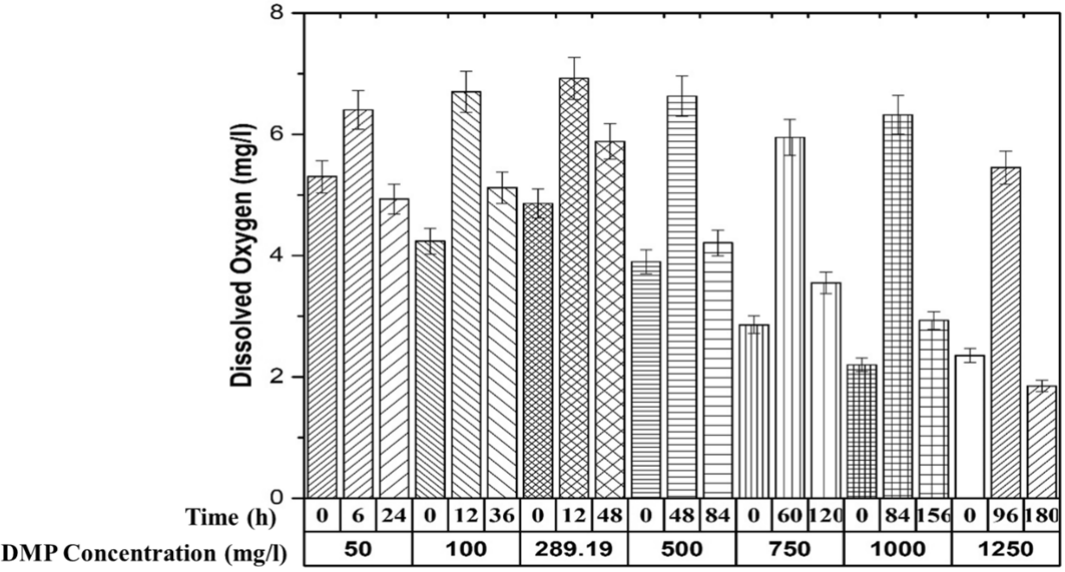
Dissolved oxygen profile during DMP degradation by *Micrococcus* sp. KS2.

Figure 5 shows the dissolved oxygen profile for DMP degradation. It was observed that the dissolved oxygen was increased at lag phase where approximately 50% of substrate degradation was occurred. At the end of the biodegradation, dissolved oxygen utilization rate was reduced it indicates the growth rate was reduced. The drop in the DO indicates that the carbon source has been completely exhausted. At optimum DMP concentration (289.19 mg/l) highest DO (6.92 mg/l) was observed, it indicates the maximum growth was occurred at this concentration. These results are coinciding with the biodegradation experiments conducted in flasks incubated in orbital shaker. This indicates that the biodegradation of DMP is oxygen-limited. Anderlei et al.^39^ studied the oxygen transfer rate for yeast culture in stirred bioreactors with exhaust gas analysis and observed similar type of behaviour. Such kind of study is not yet reported for DMP biodegradation in literature and thus, the present study is the first to execute the same.

### Growth kinetics modeling for *Micrococcus* sp. KS2

**Figure 6:**
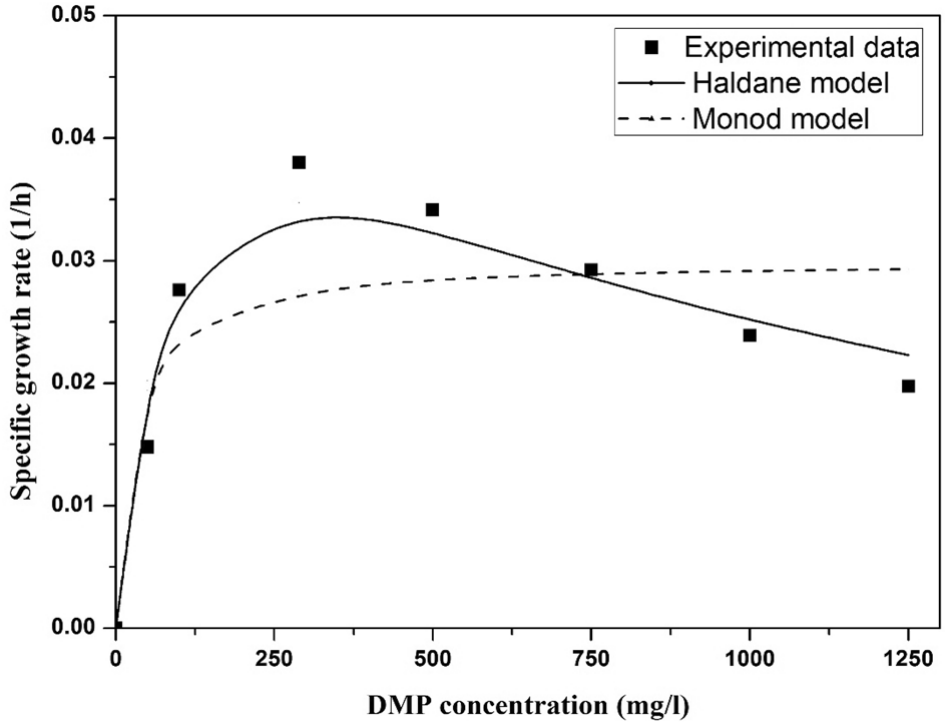
Experimental and model predicted specific growth rates of the *Micrococcus* sp. KS2 at various DMP concentrations.

The experimental data for growth of isolate was fitted with the Monod and Haldane model. Figure 6 exhibits growth kinetics of *Micrococcus* sp. KS2 for DMP degradation. It was observed that specific growth rate increases with the increase in DMP concentration up to 289.19 mg/l, but subsequently decreases, which suggest the substrate inhibition. The coefficient of correlation (R^2^) was found 0.91 and 0.76 for Monod and Haldane model respectively. It indicates that Haldane model fits well with the experimental specific growth rate. For Monod model, µ_max_, and Ks were 0.0299 h^− 1^ and 42.22 mg/l while for Haldane model µ_max_, Ks and K_i_ were 0.0596 h^− 1^, 55.38 mg/l and 388.78 mg/l respectively. The low µ_max_ for Haldane model might be due to the higher DMP concentration used in the batch biodegradation study. The Ks value point to the minimum substrate concentration that microorganism can utilize for growth^40^. Substrate inhibition constant (K_i_) was estimated high for DMP degradation by *Micrococcus* sp. KS2. Zeng et al.^41^ studied DMP degradation by *Sphingomonas* sp. strain PA-02 and reported K_i_ of 154.3 mg/l. It designates isolate having higher DMP tolerance.

## Conclusion

The present research work delivered a perspective on the biodegradation of Dimethyl phthalate (DMP) by *Micrococcus* sp. KS2. To enhance the DMP biodegradation and lessen its expenses, this process was statistically optimized. Among ten important variables, Plackett–Burman design evidenced the significance of pH, temperature, and DMP concentration. CCD was employed to optimize these significant variables and to develop a quadratic model. The optimum levels of each variable predicted from CCD are as follows: pH 7.05, temperature 31.5 °C and DMP 289.19 mg/l. It indicates the *Micrococcus* sp. KS2 is neutrophilic and mesophilic. The predicted response (99.67%) found to agree well with the experimentally obtained response (99.62%) at the optimized conditions. *Micrococcus* sp. KS2 completely degraded DMP up to 1250 mg/l within 180 h and exhibited high DMP degradation capability. Based on dissolved oxygen variation in DMP degradation by *Micrococcus* sp. KS2, it was confirmed as oxygen was limiting factor for this process. Haldane model fitted well with the experimental data for batch biodegradation. ESI–MS allowed identification of MMP and PA as a degradation intermediates. The optimum parameters and biokinetic constants obtained in this study provide the basis for further investigation of large-scale DMP biodegradation with *Micrococcus* sp. KS2 in a bioreactor. The outcomes of the present study indicated that treatment of DMP containing effluent by *Micrococcus* sp. KS2 offers a novel approach to undertake the pollution burden of PEs. The future research can focus on the detailed analysis of genes and enzymes responsible for PEs degradation by *Micrococcus* sp. KS2 in order to get an elaborative understanding of the biochemical mechanism.

## Data Availability

The datasets generated and/or analysed during the current study are available in the [GenBank of Nucleotide database of NCBI] repository, [Accession number: KY657576].

## List of symbols

Y: DMP degradation (%)
R_0_: Model intercept
R_i_: Factor linear coefficient
R_ii_: Quadratic coefficient
R_ij_: Interaction coefficient
X_i_: X_j_, Input variables
μ: Specific growth rate (h^−^^1^)
${x}_{0}$: Initial biomass (mg/l)
$x$: Final biomass (mg/l)
t: Time (h)
µ_max_: Maximum specific growth rate (h^−^^1^)
K_s_: Substrate half-saturation coefficient (mg/l)
K_i_: Substrate inhibition constant (mg/l)
